# A Transcriptomic Study of the Tail Fat Deposition in Two Types of Hulun Buir Sheep According to Tail Size and Sex

**DOI:** 10.3390/ani9090655

**Published:** 2019-09-05

**Authors:** Hongying Fan, Yali Hou, Goutam Sahana, Hongding Gao, Caiye Zhu, Lixin Du, Fuping Zhao, Lixian Wang

**Affiliations:** 1 Key Laborary of Animal Genetics, Breeding and Reproduction (Poultry) of Ministry of Agriculture, Institute of Animal Science, Chinese Academy of Agricultural Sciences, Beijing 100193, China; 2 Key Laboratory of Mariculture, Ocean University of China, Qingdao 266000, China; 3 Beijing Institute of Genomics, Chinese Academy of Sciences and University of Chinese Academy of Sciences, Beijing 100101, China; 4 Center for Quantitative Genetics and Genomics, Department of Molecular Biology and Genetics, Aarhus University, 8830 Tjele, Denmark

**Keywords:** Chinese indigenous sheep, tail type, fat metabolism, RNA-Seq, sex difference

## Abstract

**Simple Summary:**

Based on tail types, Hulun Buir sheep were divided into two lines including small and big fat-tailed, but these two lines have similar genetic background. In this study, we investigated the morphology and transcription level differences of tail fat between these two lines. The RNA-seq analyses indicated several differentially expressed genes when compared between sexes or two tail sizes. Interestingly, we also found an obvious sex difference in the fat metabolism in Hulun Buir sheep. Two different co-expression networks were only shown either in male or in female sheep. Our findings will provide theoretical background in understanding the genetic mechanism of fat deposition in sheep.

**Abstract:**

Hulun Buir sheep of similar genetic background were divided into two lines based on tail types: Small- and big fat-tailed. To explore the molecular mechanism of fat deposition in sheep tails, we firstly evaluated the morphology and transcription level differences of tail fat between these two lines. RNA-Seq technology was used to identify differentially expressed genes (DEGs) in phenotypic extremes of tail sizes. Five comparisons were performed taking into account two factors, sex and tail type. We screened out 373 DEGs between big-tailed and small-tailed Hulun Buir sheep, and 775 and 578 DEGs between two types of tails in male and female sheep, respectively. The results showed an obvious sex difference in the fat metabolism in sheep based on gene ontology (GO), pathway, and network analyses. Intriguingly, there were two different co-expression networks only respectively shown in male and female sheep, which were insulin-related network acting on upstream pathways and PPARG-related network effect in downstream pathways. Furthermore, these two networks were linked by a classic pathway of regulating adipogenesis. This is the first study to investigate the sex differences of fat metabolism in domestic animals, and it demonstrates a new experimental way to study fat metabolism. Our findings will provide theoretical background in understanding the tail-size phenotype in sheep and can be exploited in breeding small-tailed sheep.

## 1. Introduction

It is well known that distributions of body fat influence the state of human health. Fat tends to be accumulated on the buttocks in women vs. in the abdomen in men. One perspective from the Tulane University School of Medicine suggest that sex differences in metabolism should be studied in both males and females as physiological systems are not fundamentally the same in two sexes [1]. Previous reports [2,3,4,5] have explored many sex differences, such as differences in physiological processes, hormone levels, and gene expressions, and showed that fat deposit on the hips and thighs of women is healthier than around the stomachs of men. This indicated that unique genes controlled fat deposition in specific positions. Therefore, the genetic mechanism of fat deposition in specific positions and between two sexes would be different.

In sheep, fat is deposited in specific positions including tails or rumps. Sheep can be classified into five types based on size of the tail: Short fat-tailed sheep, long fat-tailed sheep, short thin-tailed sheep, long thin-tailed sheep, and fat-rumped sheep [6]. When feed is abundant, fat is stored in the tails or rumps that can be used as a source of energy for times of increased energy expenditure [7,8]. Therefore, tail fat is an important storehouse of energy for acclimatization to local, sometimes harsh, environmental conditions and feed supply fluctuations. The sizes of the tails vary both between and within sheep breeds [6,9,10].

The fat-tailed or fat-rumped sheep have been generated through a long breeding history after both natural and artificial selection. In the past, when food was scarce, fat-tailed sheep had been developed as man preferred foods that were high in calories [11,12]. However, in recent times, high-protein and low-fat foods are more likely to be selected by consumers because the consumption of high-fat foods is associated with many diseases like cardiovascular complications [13], bowel cancer [14,15], diabetes [16,17], liver disease [18,19] etc.

Hulun Buir sheep, a Chinese indigenous sheep breed, has two types of tails. One is referred to as the “small fat-tailed” line (STH). Another is Hulun Buir big fat-tailed sheep, which is also called the “Barag” line (BTH). Both these types of Hulun Buir sheep belong to the fat-tailed type. However, the tail of the Barag line is significantly larger than that of the STH line [9]. Therefore, Hulun Buir sheep is a useful model to study the molecular mechanism of fat deposition in two sexes and in tail.

RNA sequencing is a high-throughput next-generation sequencing technology to survey, characterize, and quantify the transcriptome of a tissue. It provides a genome-wide sequence readout of a transcriptome. The objective of this study was to investigate the differences in gene expression affecting fat metabolism in adipose tissue in Hulun Buir sheep with different tail sizes and sexes using RNA sequencing (RNA-seq) technology. Our findings uncover the genetic mechanism underlying fat deposition in sheep tail, and the information can be exploited in future breeding in sheep.

## 2. Materials and Methods

### 2.1. Ethics Statement

All of the animal experimental procedures were performed in strict accordance with the guidelines proposed by the China Council on Animal Care and the Ministry of Agriculture of the People’s Republic of China. All of the animal experiments were approved by the Chinese Academy of Agricultural Sciences (CAAS, Permit Number: 2014-0035).

### 2.2. Sample Collection

One hundred and fifty individuals each were selected from small- and big-tailed lines from ~2000 Hulun Buir sheep at approximately 6-month age. All these sheep were maintained under grazing condition in Hulun Buir grassland in Inner Mongolia Autonomous Region, China. Their appearances and shapes completely conformed to breed characteristics of Hulun Buir sheep and had healthy body conditions. Unrelated animals (as far as possible) with birth date records were sampled. Tail fat weights were measured after slaughter and about 8 mL of blood was collected in an EDTA-containing vacutainer and stored in liquid nitrogen (−196°). According to the tail fat weights, six sheep from each of the two lines (12 in total) were selected with extremes of the phenotypic values. These 12 sheep can be further classified into 4 groups (3 in each) based on tail types and sex: Female big-tailed Hulun Buir sheep (FBT), male big-tailed Hulun Buir sheep (MBT), female small-tailed Hulun Buir sheep (FST), and male small-tailed Hulun Buir sheep (MST). The tail fat tissues were elaborately resected from these 12 sheep. All tissue samples were stored in RNAlater™ stabilization solution (Ambion, Austin, TX, USA) at −80 °C before RNA extraction. 

### 2.3. DNA Extraction and Genotype Data

Genomic DNA was extracted from the whole blood samples using the DNeasy Blood and Tissue Kit (Qiagen, Duesseldorf, Germany). The extracted DNA was diluted to 50 ng/uL for the Ovine Infinium HD SNP BeadChip genotyping.

Three hundred sheep, including 12 sheep selected for RNA-seq, were genotyped using the Ovine Infinium HD SNP BeadChip (Illumina Inc., San Diego, CA, USA), which contained a total of 606,006 SNPs distributed throughout the domestic sheep (*Ovis aries*) genome. Firstly, a quality control on these SNPs was performed using PLINK software [20]. SNPs or individuals were removed according the criteria as Zhang et al. [10]. After the filtering, a total of 526,225 SNPs was retained in 288 individuals containing 142 small-tailed and 146 big-tailed individuals.

### 2.4. RNA Isolation and Quality Assessment

The total RNAs from tail adipose tissues were extracted using the TRIzol reagent (Invitrogen, Carlsbad, CA, USA). DNA contamination was removed using RNase-free DNase I (New England Biolabs). The sequencing library for mRNA was constructed with an IlluminaTruSeq™ RNA Sample Preparation Kit (Illumina, San Diego, CA, USA). The detailed procedures are as follows. The mRNA was purified from total RNA using poly-T oligo-attached magnetic beads and further fragmented. The cleaved RNA fragments were converted into cDNA using reverse transcription and random primers. DNA polymerase I and RNase H subsequently generated second-strand cDNA. Exonuclease/polymerase activities were used to convert remaining overhangs to blunt ends and to remove enzymes. After adenylation of 3′ ends of DNA fragments, Illumina PE adapter oligonucleotides were ligated to prepare for hybridization. To select cDNA fragments approximately 200 bp in length, the library fragments were purified with AMPure XP system (Beckman Coulter, Beverly, MA, USA). The synthesized cDNA was subjected to end repair; a single ‘A’ nucleotide overhang was attached to the 3′ end; and ligation with adapters and enrichment of cDNA fragments were performed according to the manufacturer’s instructions. RNA purity was checked using a NanoPhotometer^®^ spectrophotometer (NanoPhotometer® spectrophotometer (Implen, West Lake Village, CA, USA). RNA concentrations were measured using a Qubit^®^ 2.0 Fluorometer (Life Technologies, Carlsbad, CA, USA). RNA degradation, contamination, and integrity were checked in 1% agarose gels with a Bioanalyzer 2100 system (Agilent Technologies, Santa Clara, CA, USA).

### 2.5. RNA Sequencing and Read Alignment

The qualified cDNA library was sequenced on the Illumina HiSeq 2000 platform (Illumina, San Diego, CA, USA), producing 22~35 million clean paired-end reads with a read length of 100 bp on average for each individual. The sheep reference genome of *Ovis aries* (Oar_v3.1) was downloaded from the UCSC (http://genome-asia.ucsc.edu/cgi-bin/hgGateway?redirect=manual&source=genome.ucsc.edu) and further indexed using the Bowtie algorithm [21]. The paired-end clean reads for each sample were aligned to the reference genome using TopHat v2.0.0 (http://ccb.jhu.edu/software/tophat/index.shtml) with default options. The detailed alignment information included clean reads, Q20 and Q30 values, GC contents, error rates, and the mapping rate of total mapped reads.

### 2.6. Identification of Differentially Expressed Genes

The expression of each gene was characterized based on reads per kilo base of transcript per million mapped reads (RPKM) values [22]:

$$RPKM=\frac{10^{9}C}{NL}$$

where *C* is the number of reads mapped to gene sequence, *N* is the total number of mappable reads of a sample, and *L* is length of the gene sequence. The differentially expressed genes (DEGs) between different groups were identified using both Cufflinks [23] and DESeq [24] methods. Under the Cufflinks method, the aligned reads were first assembled with Cufflinks [23], and DEGs were identified and quantified with Cuffdiff, which is included in the Cufflinks package [23]. A negative binomial distribution was introduced for gene expression (counts) modeling for single-isoform genes, along with a mixed model of negative binomials using the beta distribution parameters as the mixture weights, followed by a *t*-test for defined statistics, to identify significant DEGs [24]. The gene expression results for different groups, including fold changes (on a log2 scale) and corresponding *p* and *q* values (false discovery rate corrected *p* values) are reported in the output files from Cuffdiff. Genes were considered differentially expressed when the *p* value was less than the false discovery rate after Benjamini–Hochberg correction for multiple testing (http://cufflinks.cbcb.umd.edu/manual.html).

Under the DESeq method, DEGs were detected using the DESeq R package (1.8.3) [24]. DESeq allows accurate comparisons between different groups by normalizing the number of reads, which accommodates a scaling factor for a given sample by computing the median of the ratio for each gene of its read count over its geometric mean across all samples [25]. A negative binomial distribution was introduced to address gene expression (counts) modeling, and Fisher’s exact test was adopted to test the significant DEGs. A *p* value of 0.05 was set as the threshold for significantly differential expression.

### 2.7. GO and Gene Function Analyses of Differentially Expressed Genes

To describe biological functions of the differentially expressed genes, we conducted gene enrichment analysis with gene ontology (GO). The Goseq R package [26] was employed for GO enrichment analysis of DEGs, in which gene length bias was corrected. GO terms with corrected *p* values of less than 0.05 were considered to be significantly enriched for the DEGs. Additionally, the ingenuity pathways analysis (IPA) software v9.0 [27] (QIAGEN Inc., https://www.qiagenbioinformatics.com/products/ingenuity-pathway-analysis) was used to evaluate the DEGs among the four groups of sheep classified based on sex and tail-fat phenotype. Firstly, the accessions of these genes were imported into IPA, and the “Core Analysis” function included in IPA was then used to analyze the genes in the context of networks, biological functions, and canonical pathways.

### 2.8. Validation of Gene Expression Using Quantitative Real-Time PCR

To validate the repeatability and reproducibility of the RNA sequencing data, quantitative real-time PCR (qRT-PCR) was carried out to evaluate the expression levels of seven DEGs related to fat. These genes were randomly selected and included UDP-glucose ceramide glucosyltransferase (*UGCG*), perilipin 4 (*PLIN4*), protachykinin-1 precursor (*TAC1*), MID1 interacting protein 1 (*MID1IP1*), lipoprotein lipase (*LPL*), Thy-1 cell surface antigen (*THY1*), and iroquois homeobox 3 (*IRX3*). The gene-specific primers were designed based on NCBI reference sequences using Primer5 software (Premier Biosoft International) and are listed in Supplementary Table S1. The glyceraldehyde-3-phosphate dehydrogenase (*GAPDH*) gene was chosen as the internal standard to adjust the input of cDNA and to normalize the expression of target genes [28]. RNA samples were obtained from the BTH and STH groups (with six replicate samples for each group). The first strand of cDNA was synthesized from 1 μg of RNA using an M-MLV Reverse Transcription Kit (Promega, USA). All cDNA products were then diluted to 200 ng/μL, and amplification reactions were prepared using SYBR^®^ Premix Ex Taq TM (Perfect Real Time) (Takara Bio, Otsu, Shiga, Japan). Each 50 μL qRT-PCR mixture consisted of 2 μL of template cDNA, 0.5 μL of each primer, 10 μL of SYBR^®^ Premix Ex TaqTM (2×), and 7.0 μL of ddH_2_O (sterilized distilled water). PCR amplification was performed in a 96-well optical plate with the following program: 95 °C for 2 min, followed by 40 cycles of 95 °C for 15 s and 60 °C for 15s. The qRT-PCR was performed using the Applied Biosystems 7500 Fast Real-Time PCR System, and the 2^−ΔΔCT^ method was employed to analyze the expression levels of genes [29]. The qRT-PCR experiments were carried out in triplicate for each sample and the average Ct value was used for further analyses. To compare with sequencing data results, log2-transformed fold-change was also calculated for qRT-PCR in each comparison. The correlation coefficients between the fold changes in the RNA-Seq and qRT-PCR groups were calculated using SPSS13.0 (SPSS Inc., Chicago, IL, USA).

### 2.9. Data Availability

The raw RNA-seq data used in this study are deposited in NCBI Sequence Read Archive (SRA, http://www.ncbi.nlm.nih.gov/bioproject/517348) under accession number PRJNA517348.

## 3. Results

### 3.1. Structure Analysis

We used the Ovine Infinium HD SNP BeadChip genotype data of 288 individuals from two lines of Hulun Buir sheep to performed unsupervised admixture analysis using ADMIXTURE 1.3 software. Results showed that they have nearly same genetic background because the individuals from two lines did not cluster into two separate groups (see Supplementary Figure S1).

### 3.2. Statistical Analysis of Tail Fat Weights

Twelve individuals from two lines were selected based on extreme phenotypes for RNA-seq study. Descriptive statistics of tail phenotypes in the two Hulun Buir sheep lines for both male and female are present in Table 1. The average tail weight of the big-tailed line sheep was 3.59 times heavier than that of small-tailed line animal (Table 1).

### 3.3. Sequencing and Mapping of Sheep Tail Fat Transcriptome

RNA-Seq was performed on 12 adipose tissues samples from sheep from the two different tail types (STH and BTH). The details on RNA qualities were showed in Supplementary Table S2. Table 2 shows the basic statistics for RNA-seq reads generated from tail fat per Hulun Buir sheep. In total, we obtained 65.98 Gb clean paired-end reads. The average GC content was 48.35 ± 1.13. The results of matching the sequencing reads against Oar_v3.1 indicated 76.1–85.6% uniquely aligned reads from the 12 samples. The average of percentage of reads that could be mapped to the reference genome of sheep was 82.88% ± 2.84. Therefore, most of the reads can be compared with exon regions, with only a small percentage of the reads showing no comparisons in gene exon regions, possibly because of incomplete gene annotations. 

### 3.4. Identification of Differentially Expressed Genes between the BTH and STH Groups

Table 3 presents the number of differentially expressed genes identified by two methods (DEseq and Cuffdiff) between BTH vs. STH. In total, 651 DEGs were determined to be significantly different by Cuffdiff, while DEseq indicated 1849 DEGs. A total of 373 DEGs overlapped between the two methods (*p* < 0.05, FDR *q* < 0.05) (Table 3). Among these genes, 11 were related to fat metabolism, including *ACSL1*, *PLIN1*, *ELOVL5*, *ELOVL6*, *ACACA*, *PTRF*, *PDK4*, *ACLY*, *ACADL*, *AGTR1,* and *EGR1*. The detailed information of the overlapping DEGs related to fat metabolism were shown in the Supplementary Table S3.

### 3.5. Gene Ontology Enrichment and Pathway Analysis between the BTH and STH Groups

To investigate the functions of the 373 DEGs, a GO analysis was performed using the GO database according to three factors: Cellular component (CC) terms, molecular function (MF) terms, and biological process (BP) terms. Among these categories, most DEGs were enriched in the BP term; however, there were only a few genes related to fat metabolism and no CC terms were found (Supplementary Table S4).

KEGG pathway analysis showed that the DEGs were involved in 30 categories. However, only three pathways were related to fat metabolism, namely fatty acid metabolism (oas01212), PI3K-Akt signaling pathway (oas04151), and regulation of lipolysis in adipocytes (oas04923). In total, 6, 14, and 5 genes were involved in these three pathways. The details on the genes and their functional annotations were provided in the Supplementary Table S5.

Next, the possible functions of the DEGs analyzed using the IPA identified 24 pathways related to lipid metabolism. According to these results, the highest number of genes (40 genes) was associated with the “synthesis of lipid” pathway (Supplementary Table S6).

### 3.6. Differentially Expressed Genes Contribute to the Sex Difference in Fat Metabolism

Moreover, Table 3 also presents the number of differentially expressed genes identified by two methods between MBT vs. MST, FBT vs. FST, MBT vs. FBT and MST vs. FST. First, the comparison of MBT vs. MST revealed a total of 7856 annotated genes differentially expressed according to Cuffdiff vs. 1117 DEGs according to DESeq, with 775 overlapping genes (Table 3) (Supplementary Table S7). Second, the results revealed 3835 and 799 DEGs between FBT and FST according to the two methods, along with 578 overlapping genes (Supplementary Table S8). Third, the results of Cuffdiff and DESeq for two sexes of the big-tailed sheep revealed 199 373 genes respectively, with 47 genes overlapped (Supplementary Table S9). Finally, comparison between MST and FST identified 491 and 164 DEGs, by two methods, respectively, with 109 genes overlapped (Supplementary Table S10).

### 3.7. Gene Ontology Enrichment and Pathway Analysis of Sex Difference in Fat Metabolism

Sex differences in fat metabolism were further evaluated to determine the functional mechanisms of the DEGs in different groups, and GO analysis was employed using the GO database. Many significant GO categories were enriched (*p* < 0.05), including several GO processes related to lipid metabolism. We analyzed the mutual relationships with fat metabolism among the MBT, MST, FBT, and FST groups. There were no genes enriched in the CC term. The largest number of genes was annotated to BP term, and a small number were annotated to MF term. Firstly, when the difference between big-tailed fat and small-tailed fat male sheep were explored, there were no GO terms related to lipids found (Supplementary Table S11). When we compared the relationship of fat metabolism between two groups of fat-tailed female sheep, no fat-related terms were found (Supplementary Table S12). However, when compared between two sexes within the same tail size, 9 biological processes were identified for big-tailed sheep, while 12 biological processes and 4 molecular functions were identified for small-tailed sheep (Supplementary Tables S13 and S14). 

Another approach for investigating the function of DEGs is KEGG pathway analysis. Diverse results were obtained through analyses of combinations of the four groups based on the tail types and sexes. First, 13 KEGG pathways with significant matches were assigned to the male sheep group with different tail sizes. Among these pathways, three terms related to fatty acid metabolism were identified, namely fatty acid degradation (oas00071), fatty acid metabolism (oas01212), and fatty acid elongation (oas00062) (Supplementary Table S15). In the second step, the female sheep were grouped according to tail size. We obtained 51 KEGG pathways in total, three of which were associated with fat, including fatty acid metabolism (oas01212), regulation of lipolysis in adipocytes (oas04923), and the PI3K-Akt signaling pathway (oas04151) (Supplementary Table S16). Again, we performed additional analysis to further elucidate the probable functional status of DEGs in the same tail-type of sheep within a sex. A total of one and five KEGG pathways were identified from the analysis of big-tailed sheep and small-tailed sheep, respectively, separated by sex (Supplementary Tables S17 and S18). According to the above results, only one pathway was connected with fat metabolism in the small-tailed sheep group, the PI3K-Akt signaling pathway (oas04151).

Finally, the possible functions of the DEGs were analyzed via IPA. The top diseases and functions associated with the DEGs were collected from four comparisons: MBT vs. MST, FBT vs. FST, MBT vs. FBT, and MST vs. FST. The results regarding lipid metabolism were as follows: 8 pathways were identified in the first comparison, 16 in the second, 19 in the third, and 8 in the fourth.

**Figure 1:**
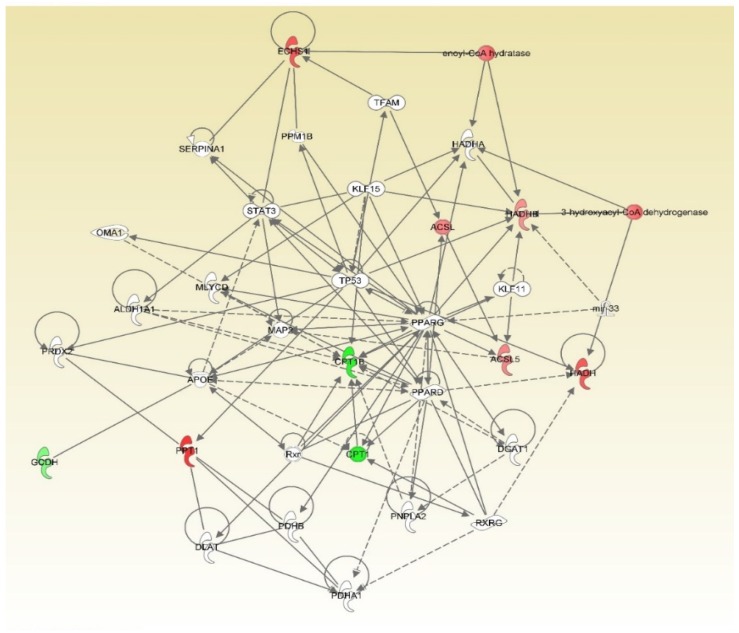
Mechanistic network for the seven fat metabolism genes. The network was generated through the use of ingenuity pathways analysis (IPA) (QIAGEN Inc., https://www.qiagenbioinformatics.com/products/ingenuity-pathway-analysis) [27]. These genes include *CPT1B*, carnitine palmitoyltransferase 1B; *ECHS1*, enoyl-CoA hydratase, short chain 1; *PPT1*, palmitoyl-protein thioesterase 1; *HADH*, hydroxyacyl-CoA dehydrogenase; *HADHB*, hydroxyacyl-CoA dehydrogenase trifunctional multienzyme complex subunit beta; *ACSL5*, acyl-CoA synthetase long-chain family member 5; *GCDH*, glutaryl-CoA dehydrogenase. In the figure, up-regulation is indicated in red and down-regulation in green.

**Figure 2:**
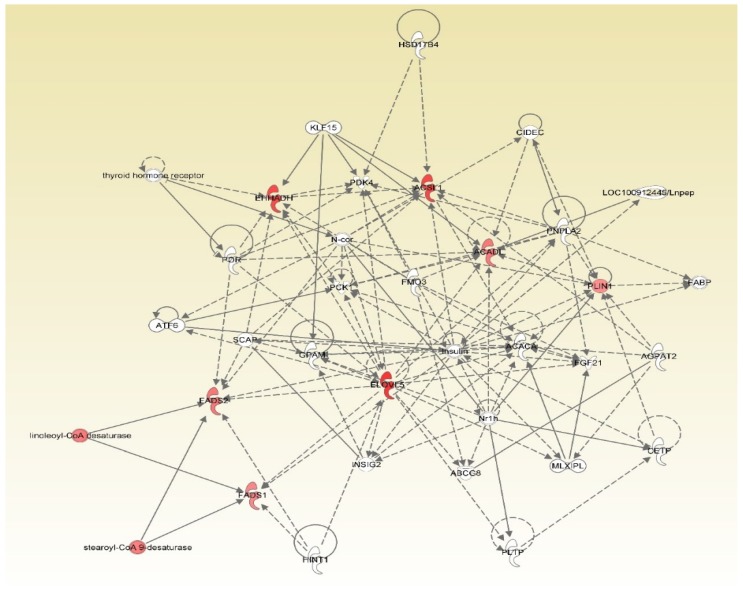
Mechanistic network of the seven fat metabolism genes. The network was generated through the use of IPA (QIAGEN Inc., https://www.qiagenbioinformatics.com/products/ingenuity-pathway-analysis) [27]. These genes include *PLIN1*, perilipin 1; *FADS1*, fatty acid desaturase 1; *FADS2*, fatty acid desaturase 2; *ELOVL5*, ELOVL fatty acid elongase 5; *ACADL*, acyl-CoA dehydrogenase long chain; *ACSL1*, acyl-CoA synthetase long-chain family member 1; *EHHADH*, enoyl-CoA hydratase and 3-hydroxyacyl CoA dehydrogenase. In the figure, up-regulation is shown in red.

It is worth noting that two networks of lipid metabolism were identified only in one of the sexes. Peroxisome proliferator-activated receptor gamma (PPARG)-related network was annotated from the comparison of big-tailed and small-tailed male sheep (Figure 1). In this network, seven genes are postulated to directly and indirectly affect PPARG and a number of other regulators, thereby explaining the changes in the expression of genes related to fatty acid metabolism in the tail fat of male Hulun Buir sheep. As shown in Figure 1, five of them, including *ECHS1*, *HADHB*, *PPT1*, *HADH,* and *ACSL5* were up-regulated while two, *CPT1B* and *GCDH,* were down-regulated. An insulin-related network was detected from the comparison of big-tailed and small-tailed female sheep (Figure 2). In this network, all seven genes, *FADS1*, *FADS2*, *PLIN1*, *ELOVL5*, *ACADL*, *ACSL1,* and *EHHADH* showed up-regulation (Figure 2). These genes are assumed to exhibit indirect or direct action in response to insulin. This can explain the differential expression of genes related to fatty acid metabolism in the tail fat between big- and small-tailed Hulun Buir female sheep. Intriguingly, the core genes in these two networks were on the classic fat metabolism pathway that regulates the adipogeneisis [30]. 

### 3.8. Validation of RNA-Seq Results by qRT-PCR

**Figure 3:**
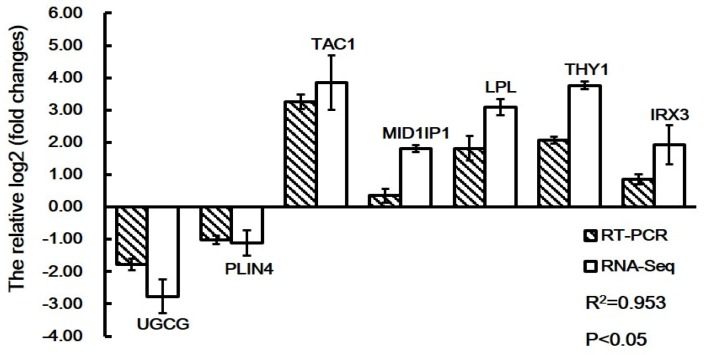
qRT-PCR validation of seven differentially expressed genes identified from RNA-seq results in the tail fat of Hulun Buir sheep. The expression levels of the selected genes were normalized to the *GAPDH* gene. Gene abbreviations are as follows: *UGCG*, UDP-glucose ceramide glucosyltransferase; *PLIN4*, perilipin 4; *TAC1*, protachykinin-1 precursor; *MID1IP1*, MID1 interacting protein 1; *LPL*, lipoprotein lipase; *THY1*, Thy-1 cell surface antigen; *IRX3*, iroquois homeobox 3.

To evaluate the reliability of RNA-seq results, seven DEGs related to fat deposition were randomly selected for qRT-PCR analysis (*n* = 6 for each line). The magnitude of fold change obtained by RNA-seq and qRT-PCR was slightly different in some instances. This validated the accuracies of the RNA sequencing data, which was verified by the qRT-PCR data (Figure 3). The Pearson’s correlation coefficient suggested that the data obtained from RNA-seq had a high significant correlation with that obtained from qRT-PCR (R = 0.953, *p* < 0.01). These results suggested that the expression profile determined by RNA-seq was reliable.

## 4. Discussion

Local shepherds have bred Hulun Buir sheep for the past centuries in the Hulun Buir grassland, a world-renowned highland pasture in an arid and semi-arid region of north China. The tail-fat is used to store energy in summer and autumn and then to provide necessary energy during cold winters, helping sheep to survive in the harsh environments. The tail-fat of sheep plays a role similar to a camel’s hump [31,32]. Hulun Buir sheep are generally classified into two categories based on tail size: Small-tail and big-tail. However, the admixture analysis with HD genotypes showed similar genetic background for both lines. In this study, individual animals of the same age and from the same region were selected, but they differed in tail types. The different regulations of gene expression related to the fat deposition observed in this study might be associated with differences in fat tail types and/or sex. The results of differential gene expression will be valuable for future studies on identification of the genes that affect fat deposition in tails.

The RNA-seq technology is a powerful approach for identification the expression levels of thousands of genes simultaneously in a tissue. The qRT-PCR analysis suggested that the transcriptome profile determined by RNA-seq were reliable. In this study, the average of percentage of reads (82.88%) that could be mapped to the reference genome of sheep was higher than 77.7% reported by Li et al. [33] and 75.6% by Kang et al. [34] and lower than 86% reported by Miao et al. [35] in adipose tissues in sheep. In other tissues of sheep, the mapping rates showed differences: 85.3%~87.7% in the longissimus muscle [36], approximately 82% in Ovaries [37], 88.10% in milk [38], and more than 70% in the intestinal regions [39].

In this study, RNA-Seq approach identified 373 DEGs between big-tailed sheep and small-tailed sheep regardless of sex. Our next analysis of fat metabolism independent of different tail sizes and types also reflected sex differences, with results revealing 775 and 578 DEGs in the male and female sheep groups, respectively. It can be deduced from these results that there was a greater number of DEGs by sex than by tail type. That is to say, sex-differential genes may be related to various biological systems, from gene expression and regulation to evolution. Fat metabolism was further examined all comparisons between different sexes and tail types. We found that the comparative analysis of different sexes in big-tailed and short-tailed sheep exhibited a total of 47 and 109 DEGs, respectively, which are smaller numbers than all of the above results. Thus, we did not identify many DEGs related to sex control based on examination of the adipose tissue in the sheep tail.

There are several genes among the DEGs associated with fat deposition, adipogenesis, fatty acid biosynthesis, and lipid metabolism in ruminant animals. *ACSL1* is a crucial gene for the lipid metabolism contributing to fatty acid biosynthesis, transport, storage, and degradation in bovine [40,41]. It was also shown that *ACSL1* was differentially regulated in the subcutaneous fat tissues of the Guangling large-tailed sheep and small-tailed Han sheep [33]. *PLIN1* is a key regulator of both basal and protein kinase A stimulated lipolysis in mammalian adipose tissue [42] and has the highest expression level in adipose tissues of cattle identified using online expressed sequence tag (EST) data [1]. In sheep milk, *ELOVL5* and *ELOVL6* were involved in the elongation of various polyunsaturated long-chain fatty acids of 18–20 and saturated and monounsaturated fatty acids with 12–16 carbons to 18 carbons, respectively [43]. *ACACA* has been showed as a potential candidate gene for fat content in sheep milk [44]. *PDK4* has been reported as related to meat quality and lipid metabolism of bovine [45]. *ACLY* is related to the lipid metabolic process and triglyceride biosynthetic process of sheep [35]. These studies suggest that *ACSL1, PLIN1, ELOVL5, ELOVL6, ACACA*, *PDK4*, and *ACLY* are plausible functional candidate genes of the metabolism regulation of tail-fat in sheep. Their functional verifications need to be conducted to validate our findings.

To gain insight into the predicted gene networks, GO and pathway enrichment analysis were performed. Comparisons between the BT group and the ST group resulted in assignment to 55 GO terms, with the male sheep groups being allocated to 40 GO terms, while 75 GO terms allocated to female groups. In summary, we obtained three identical GO terms across all sheep groups and the male groups, while 19 of the same GO terms were obtained from all sheep groups and the female groups. In addition, genes in the male and the female sheep groups did not overlap, and there were no identical GO terms between the male and female sheep with the same tail type. The above results indicate that the functional classifications of DEGs in male and female sheep are different. Previous reports in humans also suggest differences between men and women in fat deposition patterns, fat metabolism, and the health consequences of obesity [46,47,48,49]. Additionally, there are similar reports on DEGs related to fat metabolism in other species that have been associated with sex differences; for example, serum leptin levels are strongly correlated with female sex, being expressed three times higher than in males [50,51]; RXRα was found to be involved in male–female differences in the expression of lipid processing genes [52]; and support has been obtained for the hypothesis that females exhibit higher activity of the promoter of the PPARγ gene than males in visceral adipose tissue, due to retinoic acid produced by the action of Aldh1a1 [53].

KEGG pathway analysis was performed to further elucidate the probable functional status of DEGs. The results for the enrichment of the DEGs in the KEGG database showed that the total number of associated pathways was variable; nevertheless, the number of pathways related to fat metabolism was equal or similar between the groups. Total number of pathways assigned to the female sheep group was significantly higher than that assigned to the male sheep group, even though the male sheep group had more DEGs. This finding implied that the fat metabolism of female sheep could be more complex because females exhibit pregnancy, birth, and lactation behaviors. Scientists have also investigated sex differences in humans [48,54,55,56] and mice [57,58,59], but such differences have rarely been studied in livestock. Study on fat metabolism in domestic animals has generally been conducted using a simple random-sampling method and/or in a group of experimental animals with a sex distribution of 50% [35,60]. The transcriptomic study of the tail-fat in the course of pregnancy, birth, and lactation in female sheep will be carried out in the future.

The results presented above revealed only one pathway that was clearly related to fat metabolism: The fatty acid metabolism pathways. However, unexpectedly, different genes were identified in the enrichment analyses of different groups. For example, the results for the BTH vs. STH groups included *ACSL1*, *ELOVL5*, *EHHADH*, *ACACA*, *ELOVL6*, and *ACADL*; those for the male sheep groups included *CPT1B*, *ECHS1*, *PPT1*, *HADH*, *HADHB*, and *ACSL5*; while those for the female sheep groups induced *ACSL1*, *ELOVL5*, *FADS1*, *EHHADH*, *FADS2*, and *ACADL*. The results indicate that there are differences in genes in the same pathways between male and female sheep, which indicates that the regulatory mechanism of fat metabolism may be different for two sexes in sheep. Previous research has suggested that sex hormones may influence the enzymatic synthesis of long-chain polyunsaturated fatty acids and has shown sex-specific differences in some outcomes [61]. The adipose tissue of the tail of sheep is mainly composed of triglycerides [62], which is a fat molecule formed by long-chain fatty acids and glycerin.

**Figure 4:**
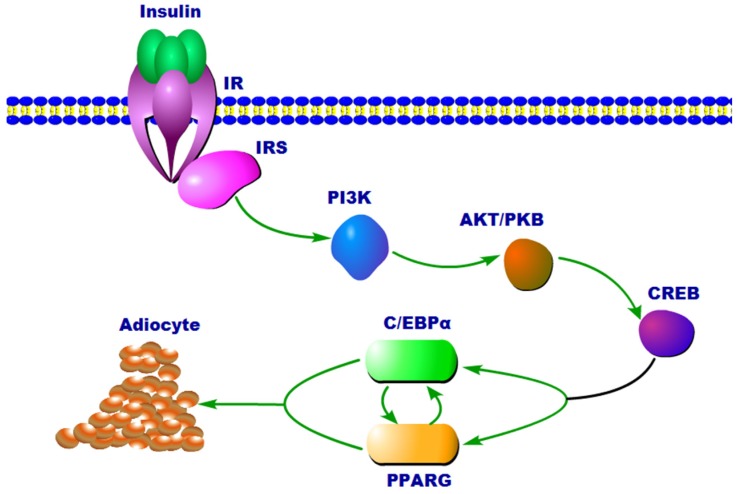
Relationship of adipogenesis signals that directly or indirectly regulate expression that are related to insulin and PPARG (peroxisome proliferator-activated receptor gamma). IR, insulin receptor; IRS, insulin receptor substrate; PI3K, phosphatidylinositol-3 kinase; AKT/PKB, serine/threonine kinase, also known as protein kinase B; CREB, cAMP responsive element binding protein; C/EBPα, CCAAT-enhancer-binding protein α.

Our findings included PPARG-related and insulin-related pathways, which are associated with fat metabolism in male sheep and female sheep, respectively. The peroxisome proliferator-activated receptors (PPARs) are members of the nuclear receptor superfamily of ligand-inducible transcription factors [63]. Three PPAR isoforms are encoded by separate genes in mammals, including *PPARA* (also known as *NR1C1* or *PPAR*α), *PPARD* (also known as *NR1C2* or *PPAR*β/δ), and *PPARG* (also known as *NR1C3* or *PPAR*γ). The PPARs control the expression of networks of genes involved in adipogenesis, lipid metabolism, inflammation, and maintenance of metabolic homeostasis [64]. Insulin plays a significant role in adipogenesis as well. Insulin can act on adipogenesis through insulin receptor substrate (IRS) signaling, promoting cAMP responsive element binding protein (CREB) phosphorylation, followed by the activation of downstream pathways, including the PPARG signaling pathway [30]. Based on the findings of Ahmadian et al. [64] and Rosen et al. [30], we simplified the classic pathway of regulating adipogenesis, as shown in Figure 4. This result presumably suggests that the differences in lipolysis in female sheep occur upstream of signal-transmitting pathway, while in male sheep they may occur in downstream pathways. The functional validation of the pathways will be carried out in future.

## 5. Conclusions

To the best of our knowledge, this is the first study to investigate the difference of fat metabolism mechanisms in two lines of Hulun Buir sheep caused by morphological and sex differences. Our study successfully identified the DEGs related to fat metabolism in five comparisons according to tail size and sex of Hulun Buir sheep. Our findings will not only help to determine the functional complexity of fat deposition in sheep, but also will provide valuable information for understanding the phenotypic and functional differences of fat deposition in tails.

## Figures and Tables

**Table 1:** Summary statistics of weights of tail fat of big- and small-tailed Hulun Buir sheep lines (kg).

Breed	Sex		Overall
	Female	Male	
Big-tailed Hulun Buir sheep	1.357 ± 0.572	1.630 ± 0.803	1.493 ± 0.641
Small-tailed Hulun Buir sheep	0.316 ± 0.057	0.515 ± 0.157	0.4153 ± 0.152

**Table 2:** The basic statistics for RNA-seq reads generated from tail fats of Hulun Buir sheep.

Mapping Summary	Big-Tailed Sheep						Small-Tailed Sheep					
	Male			Female			Male			Female		
	Sheep1	Sheep2	Sheep3	Sheep4	Sheep5	Sheep6	Sheep7	Sheep8	Sheep9	Sheep10	Sheep11	Sheep12
Cleanbases (G)	5.88	7.06	4.48	4.80	4.42	5.20	5.36	5.26	5.32	6.06	6.16	5.98
Q20 (%)	95.00	96.25	96.34	95.87	95.78	96.21	96.01	96.55	96.18	95.45	95.57	92.82
Q30 (%)	87.90	89.88	89.96	89.95	90.01	89.83	89.12	90.51	89.55	89.09	89.37	84.11
GC content (%)	49.91	49.57	49.51	47.74	45.80	47.68	48.81	47.31	48.27	48.75	48.38	48.50
Error rate (%)	0.62	0.46	0.45	0.515	0.53	0.48	0.49	0.425	0.475	0.555	0.545	0.91
Mapping rate (%)	76.10	83.00	83.10	84.60	82.50	84.50	84.10	85.60	84.50	84.00	84.30	78.20

Q20: The percentage of bases with a Phred quality score > 20%; Q30: The percentage of bases with a Phred quality score > 30%.

**Table 3:** Identification of differentially expressed genes by two methods among all groups.

Method	Group	Cutoff	DEGs
cuffdiff	BTH vs. STH	*q* < 0.05	651
cuffdiff	MBT vs. MST	*q* < 0.05	7856
cuffdiff	FBT vs. FST	*q* < 0.05	3835
cuffdiff	MBT vs. FBT	*q* < 0.05	199
cuffdiff	MST vs. FST	*q* < 0.05	491
Deseq	BTH vs. STH	*p* < 0.05	1849
Deseq	MBT vs. MST	*p* < 0.05	1117
Deseq	FBT vs. FST	*p* < 0.05	799
Deseq	MBT vs. FBT	*p* < 0.05	373
Deseq	MST vs. FST	*p* < 0.05	164
two method overlap	BTH vs. STH		373
two method overlap	MBT vs. MST		775
two method overlap	FBT vs. FST		578
two method overlap	MBT vs. FBT		47
two method overlap	MST vs. FST		109

BTH: Big-tailed Hulun Buir sheep, STH: Small-tailed Hulun Buir sheep, FBT: Female big-tailed Hulun Buir sheep, MBT: Male big-tailed Hulun Buir sheep, FST: Female small-tailed Hulun Buir sheep, MST: Male small-tailed Hulun Buir sheep, *q*: the adjust *p* value.

## Supplementary Materials

The following are available online at https://www.mdpi.com/2076-2615/9/9/655/s1. Figure S1: Population structure inferred using ADMIXTURE analysis, Table S1: Primers used for quantitative Real-Time PCR (qRT-PCR) validations between the big-tailed and small-tailed Hulun Buir sheep. Table S2: The details of RNA qualities for each sample, Table S3: The details of differential expression genes identified by two methods between all big fat-tailed and short fat -tailed sheep, Table S4: The results of GO analysis between all big fat-tailed and short fat -tailed sheep, Table S5: The summaries of KEGG analysis between all big fat-tailed and short fat -tailed sheep, Table S6: The finding of network analysis between all big fat-tailed and short fat -tailed sheep, Table S7: The details of differential expression genes identified by two methods between male big fat-tailed and short fat -tailed sheep, Table S8: The details of differential expression genes identified by two methods between female big fat-tailed and short fat -tailed sheep, Table S9: The details of differential expression genes identified by two methods between female and male sheep with big tail, Table S10: The details of differential expression genes identified by two methods between female and male sheep with small tail, Table S11: The results of GO analysis between male big fat-tailed and short fat -tailed sheep, Table S12: The results of GO analysis between female big fat-tailed and short fat -tailed sheep, Table S13: The results of GO analysis between female and male sheep with big tail, Table S14: The results of GO analysis between female and male sheep with small tail, Table S15: The summaries of KEGG analysis between male big fat-tailed and short fat -tailed sheep, Table S16: The summaries of KEGG analysis between female big fat-tailed and short fat -tailed sheep, Table S17: The summaries of KEGG analysis between female and male sheep with big tail, Table S18: The summaries of KEGG analysis between female and male sheep with small tail.
